# Flipping the Script: An Initial Exploration of Flipped Learning as an Enhanced Alternative to Traditional Physical Education Lessons

**DOI:** 10.3390/ijerph192215188

**Published:** 2022-11-17

**Authors:** Chad M. Killian, Sarah M. Espinoza, Collin A. Webster, Belden Long, Mark Urtel, Amelia Mays Woods, Emily M. D’Agostino

**Affiliations:** 1 Department of Kinesiology, University of New Hampshire, Durham, NH 03824, USA; 2 Division of General Pediatrics and Adolescent Health, University of Minnesota, Minneapolis, MN 55455, USA; 3 Exercise and Rehabilitation Sciences, University of Birmingham Dubai, Dubai P.O. Box 341799, United Arab Emirates; 4 Department of Orthopaedic Surgery, Occupational Therapy Division, Duke University School of Medicine, Durham, NC 27707, USA; 5 Department of Kinesiology, Indiana University-Purdue University, Indianapolis, IN 46202, USA; 6 Department of Kinesiology and Community Health, University of Illinois, Champaign, IL 61801, USA

**Keywords:** blended learning, digital instruction, information and communication technology, middle school

## Abstract

Background: Physical activity (PA) is related to a variety of positive outcomes for youth and physical education (PE) represents a primary school-based environment where students can engage in moderate-to-vigorous physical activity (MVPA). Limitations exist in secondary schools with low socio-economic status, where MVPA engagement is generally below the recommended 50% of lesson time. Growing evidence suggests that using a flipped learning approach (FLA) may naturally enhance PE lessons as outlined by The Theory of Expanded, Extended, and Enhanced Opportunities for Youth Physical Activity Promotion. Purpose: The purpose of this study was to compare the impact of the FLA to traditional instruction on student MVPA, lesson context, and teacher involvement during middle school PE lessons. Methods: Participants were 56 male students from two 7th-grade classes from a low-SES, suburban school. This study employed the System for Observing Fitness Instruction Time (SOFIT). Data were examined through descriptive statistics. Linear regression models were used to predict SOFIT outcomes as a function of FLA versus traditional instruction. Results: Students in the FLA class spent significantly more class time in MVPA (*p* < 0.01). Regression models predicted participants in FLA classes would spend 55% more class time in MVPA (*p* < 0.01). After adjusting for covariates, models showed students in FLA classes would spend almost 18 more minutes in MVPA than students in traditional classes (*p* < 0.01). Discussion/Conclusion: The FLA may be a practical lesson enhancement strategy to increase student MVPA and improve PE opportunities for students in low SES schools when compared to the use of traditional instruction. The results of this study demonstrated positive potential of using FLA in PE but should be considered within the context of their limitations. Further examination of the FLA is warranted.

## 1. Introduction

Physical activity (PA) has many benefits for school-aged youth, such as increased physical fitness, reduced symptoms of depression, and improved cognition. These benefits last into adulthood and can protect against obesity, high blood pressure, and depression, elevated insulin and blood lipids [1]. International consensus is that children in this age group should accumulate at least 60 min of moderate-to-vigorous PA (MVPA) each day [2], but fewer than 1 in 4 youth meet this guideline [3]. This problem is exacerbated for youth from underrepresented groups and from families classified as low socio-economic status (SES), who tend to be less active and more sedentary than their counterparts [4]. Moreover, the transition from childhood to adolescence may be marked by a decline in PA levels [5]. This period of child development, particularly the years spent in middle/junior high school [6,7], presents a critical window for intervention—particularly for youth from low-income and underrepresented groups where the research has been scant and somewhat conflicting [8].

In the United States, school physical education (PE) should provide the foundation for lifelong PA through instruction that focuses on the development of knowledge and skills needed to be a competent and confident mover [9]. PE also serves as an opportunity for students to be physically active, thus supporting the goal of helping children accrue 60 min of MVPA each day [10]. However, there is no federal law that requires PE be taught in schools; many states have policies that lead to sparsely allocated curriculum time and allowable exemptions from, or substitutions for, PE, which limits the potential of PE to optimally impact student learning or PA [11]. At the middle school level, more than one quarter (13) of the states do not require PE during middle/junior high school, and only 12 states have policies that specify the number of minutes that should be devoted to PE each week [11]. Further, there tends to be fewer MVPA opportunities during PE in secondary schools with low SES where time spent in MVPA generally falls well below the recommended 50% of lesson time in these contexts [12]. These trends echo a long-standing discourse among PE professionals who have lamented the lack of attention school leaders give to PE, which has contributed to a continuous narrative of marginalization and, ultimately, the inability of PE teachers to realize program goals [13]. Based on the available evidence, less than half of U.S. students are likely achieving national content standards [14]. Innovative strategies are needed to help PE programs increase their impact, demonstrate their efficacy, and “flip the script” about what they can accomplish.

### 1.1. Flipped Learning Approach

Working to effect policy change at higher levels of the education system to increase support for PE is a challenging and long-term process [15]. While pursuing such support must continue to be a priority for PE programs, alternative, shorter-term solutions to increasing program impact should also be considered and tested. The flipped learning approach (FLA, also referred to as the “flipped classroom”) offers one potential strategy for increasing opportunities for both student learning and PA through PE [16,17]. Østerlie and colleagues define FLA within a PE context as a pedagogical approach that uses “asynchronous digital instruction for the expansion of learning and the promotion of physical activity opportunities beyond the PE class, which is designed to enrich students’ skills and knowledge for upcoming face-to-face classes where they engage in collaborative, guided movement experiences designed to extend and deepen their prior learning” ([17], p. 113). This definition reflects how digital learning in FLA can work to offer added independent learning, which can also scaffold face-to-face learning opportunities during class. Indeed, a foundational purpose for teachers using the FLA is to increase the amount of in-person, during school class time available for active learning, without reducing the extent or quality of instructional delivery, learning tasks, or assessments (e.g., watching demonstrations of sports skills, taking cognitive assessments) [18,19,20]. This is possible and inherent to FLA format where a bulk of direct instruction should be placed online for students to review prior to class. Transferring the direct instruction online offers a natural lesson enhancement strategy by effectively removing it from face-to-face instruction, thereby creating space during class for added active learning opportunities (where the direct instruction would have normally occurred). For example, if a teacher wanted to use the FLA to help increase active learning in their classes, they might identify lessons that include more complex instructions or involve more comprehensive demonstrations, which require students to sit a listen for longer periods of time. When a teacher transfers those instructions or demonstrations online for students to review prior to class, the teacher no longer needs to take time during class to provide that content. Instead, they might conduct a brief review at the beginning of class and allow students to engage in active learning opportunities to apply their knowledge. In this way, the transition of cognitive domain content to an online format can influence psychomotor domain learning in face-to-face contexts. Because this instruction is occurring in PE, use of the FLA should result in increases in active learning in the form of added PA and skill practice opportunities. Initial evidence points to the potential of FLA format to enhance PA opportunities in PE. Thus far, one study on FLA has documented distinct increases in motor skill practice during high school PE lessons compared to traditional instruction [21]. These findings are supported by PE students and teachers who reported perceived added PA opportunities during FLA lessons [22,23,24].

Teacher and student views on FLA have been generally favorable and describe the positive impact of its use on PE student learning and motivation [24,25,26]. For example, Killian and colleagues reported that university students perceived an increase in teacher feedback when FLA was used in an individual sports course [22]. Moreover, Lucena and colleagues found that elementary and secondary students who used FLA reported higher evaluations of motivation, autonomy, and class interactions than students who used a traditional approach to PE [27]. Teachers reported how FLA helped to maximize instruction, assessment, and examination practices [23,24].

More generally, FLA has the advantage of promoting student engagement with digital technologies, which aligns with 21st century learning goals [24]. Given widespread use of online instruction throughout COVID-19 lockdowns, FLA might also offer teachers a practical way to efficiently review content by reimplementing previously developed online modules to support continued learning during regular, in-person lessons [28]. Despite evidence pointing to the potential enhancement effect FLA could have on youth PA opportunities in PE, there is a need for further research. Previous studies were mostly conducted in high school settings (9th–12th grades), and there is a paucity of relevant research in low SES and urban schools [29]. Furthermore, despite a recent focus on issues related to diversity, equity, and inclusion in PE, which has sought to inform practice within traditional, face-to-face PE environments [30], these issues have generally been overlooked within the online and FLA PE literature. Another limitation of these previous studies is that they are based on self-report and interview data. No observational studies have explored the influence of FLA on students’ PA or teacher instruction in K-12 PE settings. Direct observation of students’ PA, concurrent teacher behavior, and lesson-level variables in the context of FLA would provide initial objective data on the potential of FLA to enhance PE and promote both PA- and learning-related outcomes.

### 1.2. Theoretical Framework

The current study was based on the Theory of Expanded, Extended, and Enhanced Opportunities for Youth Physical Activity Promotion (TEO) as a way to understand the potential of FLA to positively influence MVPA, lesson context, and teacher involvement [31]. Whereas many theories applied to research on youth PA promotion focus on complex psychological and socio-cultural mechanisms of change, the TEO takes a more pragmatic approach that gives increased emphasis to PA opportunities and considers how these opportunities can be modified to increase PA engagement. Specifically, based on an extensive review of the research evidence, the TEO suggests that PA opportunities can be expanded, extended, or enhanced to increase youth PA. *Expanding* involves adding new opportunities. In the context of PE, this would mean increasing the number of lessons that students receive each week. When an opportunity is *extended*, the same opportunity is allocated more time. Thus, rather than offering additional PE lessons, the existing lessons would be lengthened. The *enhancement* of PA opportunities is defined as “strategies designed to increase physical activity above routine practice” (p. 120) [31]. Enhancing PA promotion through PE would entail optimizing the use of already available lesson time to increase students’ PA levels.

Both expanding and extending PA opportunities require scheduling changes and possibly reducing time currently devoted to other school activities to accommodate increased PE time. These PA promotion approaches may be unappealing to school leaders who choose to give little priority to PE. Furthermore, in a previous observational study comparing traditional-scheduled (52–58 min on 5 days per week) and modified block-scheduled PE lessons (57–58 min on 3 days per week and 110–130 min on 1 day per week), the extended lesson time did not result in increased levels of PA for high school students [32]. One of the factors that was negatively associated with PA engagement in that study was time devoted to developing students’ knowledge about the lesson content, such as when the teacher presents information about a learning task or gives students performance feedback. A key feature of FLA may allow teachers to address this issue. Within FLA, the traditional instruction is transferred online for students to review prior to class specifically as a means to increase active learning time (i.e., PA engagement) during class. This represents a potential natural enhancement characteristic when applied within PE. In line with the language of the TEO transitioning direct instruction online would be a “strategy designed to increase physical activity above routine practice (p. 120) [31]. This would work to optimize already available lesson time by allowing students to engage in MVPA (active learning) in place of sedentary time listening to instructions. Therefore, FLA may offer a feasible and effective way to increase students’ lesson time in MVPA without compromising attention to students’ knowledge development, changing school schedules, or requiring additional PE resources (e.g., hiring additional PE teachers to support expanded programing).

### 1.3. Purpose of the Study

Overall, there is an urgent public health need to better understand how to optimize PE as an opportunity to increase and promote PA in middle-school aged youth. The TEO, in combination with FLA, offers a practical perspective for addressing this need via enhanced PE lessons that maximize time for PA while preserving, and possibly improving, instructional processes and student learning. As current FLA research is mostly limited to self-report data, a logical next step for advancing research in this area is the use of observational methods to objectively capture teacher behavior, lesson context, and student outcomes. The purpose of this exploratory study, therefore, was to initially examine, from the perspective of TEO and through direct observation, the impact of FLA applied within a PE invasion games unit in a high-poverty, majority non-White middle school. Specifically, the study addressed the following research questions:

(1) How do levels of student MVPA differ between a FLA and a traditional, in-person instruction approach?
(2) How do lesson context and teacher involvement differ between a FLA and a traditional, in-person instruction approach?

This study is important because it considers a practical, economically viable, and teacher-led approach to increasing what PE can accomplish despite limited curriculum time in schools.

## 2. Materials and Methods

Two middle school PE classes participated in an invasion games (soccer) unit. Both classes received identical content; however, one was taught through traditional, in-person instruction while the other class learned through FLA. Systematic observations were conducted to compare student MVPA, lesson context, and teacher behavior between the teaching approaches. Approval from the University of Illinois Institutional Review Board was obtained prior to data collection.

### 2.1. Setting and Participants

A public suburban middle school (grades 6–8) located in the Midwestern United States served as the setting for this study. At the time of data collection, 95% of students in the school were classified as low income. A total of 62% of students in the school were Black, 34% were Hispanic, 3% were American Indian, and 1% were White [33]. Students in the district were allotted daily PE and were required to participate in single-sex classes during middle school. In support of recently initiated district technology integration policies, all students were issued a Google Chromebook and teachers were expected to use the Chromebooks to support learning within their subjects.

Participants in this study were 56 males from two 7th grade PE classes. Average attendance for each PE class was 27 (class attendance range over the course of observations: 22–30 students). Students had not been taught soccer as an invasion game during the school year and had never participated in the FLA in any class.

One male teacher with 35 years of teaching experience delivered all the lessons for both classes in this study. It was the first time he implemented instruction using FLA. This teacher was involved in leadership positions at state, regional, and national professional organizations and was recognized as an exemplary physical educator over the course of his career. He independently designed and implemented the content and assessments for the unit, including the digital instruction used for the FLA group.

### 2.2. Lesson Structure

The invasion games unit observed consisted of five lessons and emphasized the development of soccer skills and invasion game tactics. Both classes followed the same lesson plans and used the same instructional PowerPoint slides and assessments; however, the slides and assessments were transferred online for FLA students to review prior to class.

#### 2.2.1. Traditional Lessons

The first four traditional instruction classes occurred using standard PE practices where the teacher offered lesson content through the provision of teacher-centered, whole-class task presentations. Following large group instructions, students engaged in various skill development activities and small-sided games. During the activities, the teacher circulated around the gym to provide feedback to individuals and small groups. The final unit assessment was administered during the fifth lesson. Students completed the paper and pencil knowledge test at the beginning of the period, then were allotted free play for the duration of the class (most students chose to play basketball).

#### 2.2.2. FLA Lessons

**Figure 1:**
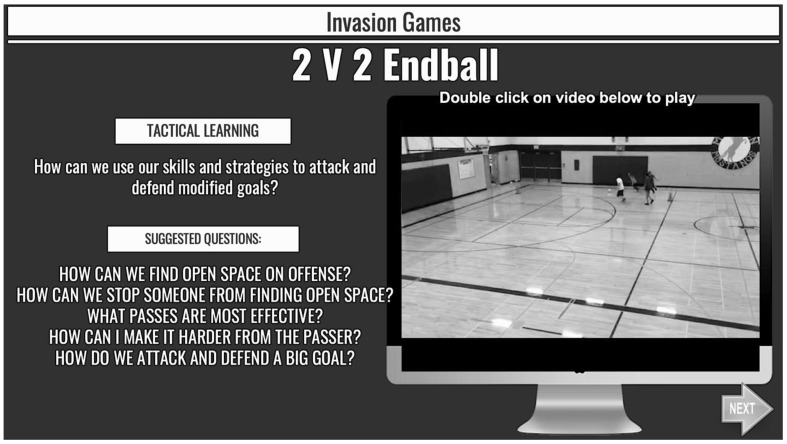
Instructional Slide Used as a Part of the FLA. Note. This figure contains the essential tactical learning question for the upcoming face-to-face lesson, as well as a video that describes and demonstrates the main applied activity students will participate in during class. The Suggested Questions represent areas of reflection and encourage students think about key tactical strategies that will be emphasized in upcoming lessons.

The FLA classes received the same content as students in the traditional instruction classes. However, they engaged with the instruction before class, online, and through text slides and teacher-curated videos in alignment with FLA implementation principles [16,34]. Digital content consisted of a 30-slide PowerPoint presentation that was made available through the schools’ Learning Management System. Slides corresponded to each in-person lesson and students were required to review them prior to the class and the entire slide deck was made available to students at the onset of the unit to allow for self-paced autonomous engagement with the content. Slides contained text related to important concepts that would be emphasized in subsequent in-person classes, video demonstrations of skills and activities they would be performing, as well as key questions for reflection (see Figure 1 for an example). Students were also required to complete a formative accountability assessment for each digital lesson. These were designed to encourage focused engagement with content prior to class.

School policy dictated that students in this school were required to gather in an assigned homeroom for 25 minutes prior to the commencement of the first class period. During this time, all students were served free breakfast and were allotted time to complete homework, study, and socialize. Policy also stipulated that homeroom classes would attend enrichment classes like PE and music together. Since students in the FLA PE class also attended homeroom together, the PE teacher coordinated with their homeroom teacher to ensure they engaged with their flipped PE content on their Chromebooks. The homeroom teacher also agreed to supervise students while they completed their formative PE lesson assessments online, including the final knowledge assessment.

### 2.3. Data Collection

Each of the traditional instruction (n = 5) and FLA lessons (n =5) was coded in real-time, on-site using the System for Observing Fitness Instruction Time (SOFIT) [35,36]. SOFIT is a validated systematic observation tool that uses alternating 10-s observe, 10-s record intervals to code student PA intensity levels, lesson context, and teacher interaction during a lesson [37,38]. The SOFIT description and procedures manual was followed for this study and provides standardized observer training protocol, operationalized definitions of coding categories, and detailed steps for observation and reliability assurance [35]. Five target students were randomly selected for observation at the beginning of each class to serve as general representatives of class activity levels. Four students were observed individually on a four-minute sequential, rotational basis for the duration of the lessons. The fifth served as an alternate in the event one of the primary target students discontinued participating. Observations began when over half of the students entered the learning space and the teacher was present in the learning space [35].

Student PA intensity was coded on a 1–5 scale based on whether the target student was *lying*, *sitting*, *standing*, *walking,* or engaged in *vigorous* PA. MVPA represents a combination of walking and vigorous PA codes. Lesson context was coded as one of six primary PE subject matter delivery systems (i.e., *general content/management*, *knowledge*, *fitness*, *skill practice*, *game play*, *free play/other*). Teacher involvement was coded using the alternate SOFIT method for assessing teacher involvement to allow for the collection of a wider range of behaviors [35]. This version expands beyond assessing teachers’ physical activity promotion to include coding across a hierarchy of categories, which include whether the teacher *promoted fitness*, *demonstrated fitness*, *instructed generally*, *managed*, *observed*, or completed *other tasks*. The category of *instructed generally* was coded as either “whole group” or “small group or individual” to investigate potential differences in instructional interactions between the two teaching approaches.

The first author served as the Lead Observer for all 10 lessons, while a research assistant served as the Reliability Observer. Both observers were trained to the gold standard as SOFIT data collectors. Initial training included following the SOFIT training manual and digital materials available online through SOFIT [35]. The training process consisted of observers spending at least two hours of studying coding definitions and five hours of coding videos of PE classes freely available online. Observers met regularly to clarify understanding of coding definitions and conventions.

Intraobserver and interobserver reliability checks were conducted at the end of training. To enhance the rigor of training reliability, observers allotted at least one-week between intraobserver checks. To maintain independence, observers refrained from reviewing or accessing the observation record from the first reliability observation session until after the second observation was conducted. Independence was achieved during the interobserver reliability checks by conducting the observations separately before meeting to review [39] Prior to data collection, the Lead Observer and Reliability Observer completed a final, independent interobserver reliability on-site, during a live class. Reliability for the trainings, interobserver checks, and study observations achieved above the recommended 85% agreement threshold across SOFIT categories which was in alignment with adequate reliability and consistent with previous SOFIT research conducted in the United States [35,40].

### 2.4. Variables of Interest, Design, and Analyses

#### 2.4.1. Main Variables

The independent variable was whether a classroom had FLA versus traditional instruction (binary variable). Each class had the same teacher, and classes were consistent across student demographics (racial/ethnic composition, socioeconomic status, and grade level).

Given this study’s focus on the FLA as a strategy for PA promotion, its two main SOFIT dependent variables were (1) percent of class time during which students participated in MVPA and (2) total minutes that students spent in MVPA. Secondary SOFIT outcomes included lesson context (operationalized as percent of class time spent on general content, knowledge, skill practice, or free play), and teacher involvement (operationalized as percent of class time spent when the teacher instructs a small group, instructs a whole group, manage, observes, or performs other tasks).

#### 2.4.2. Covariates

Class size (total students), length of class (total minutes), and classroom setting (indoors or outdoors) were included as potential confounding variables based on previous literature, which shows an association between these factors, classroom instruction approach, and school-based physical activity engagement [41,42].

#### 2.4.3. Design and Analyses

This study relied on a quantitative, cross-sectional design, as researchers measured dependent variables at the same time the teacher implemented FLA and traditional instruction approaches. As the first step in analysis, descriptive statistics were computed to summarize means and standard deviations for physical activity engagement, lesson context, teacher involvement, and covariates for all FLA and traditional instruction classes. Next, individual regression models were tested to predict each physical activity engagement, lesson context, and teacher involvement variable based on FLA versus traditional instruction. Finally, adjusted regression models were tested to predict physical activity engagement, lesson context, teacher involvement variables as a function of FLA versus traditional instruction while accounting for class size, length of class, and classroom setting. A *p*-value of <0.05 was used to determine statistical significance; all analyses were performed using SAS 9.4 software (Cary, NC, USA).

## 3. Results

### 3.1. Descriptive Statistics of Flipped and Traditional Instruction Classes

Descriptive statistics of all FLA and traditional instruction class sessions are summarized in Table 1. Class sizes, environment (i.e., inside vs. outside), and length of class were comparable between FLA and traditional instruction classes. The percentage of lesson time students spent in MVPA was higher in FLA classes (M = 74.84%, SD = 14.85%) compared to the traditional instruction classes (M = 53.12%, SD = 15.23%); FLA classes also spent more minutes in MVPA (M = 24.16 min, SD = 6.01 min) than traditional instruction classes (M = 17.20 min, SD = 5.07 min). On average, traditional instruction classes spent more time learning general content and building knowledge than FLA classes, whereas FLA classes spent more time engaged in skill practice and free play. Regarding teacher involvement, the PE teacher typically spent more time in whole group instruction and managing students when engaged in traditional instruction, whereas he spent more time in small group instruction, student observation, and completing other tasks in FLA classes. Three SOFIT variables (fitness activity, teacher promotes fitness, and teacher demonstrates fitness) were not observed in any classes. Observed classes focused on invasion games (soccer) rather than fitness.

### 3.2. Regression Results Predicting SOFIT Outcomes Based on Instructional Approach

Individual regression models predicting each SOFIT variable based on instructional approach and adjusting for covariates yielded significant findings for MVPA, lesson context, and teacher involvement. See Table 2.

#### 3.2.1. MVPA

Independently and when adjusting for class size, length of class, and whether a class took place indoors or outdoors, classes in the FLA condition spent a significantly higher percentage of class time involved in MVPA than did traditional instruction classes; accounting for covariates, regression models predicted that participants in FLA instruction classes would spend 55% more class time in MVPA than students in traditional instruction classes (*p* = 0.009). After adjusting for covariates, regression models indicated that students in FLA classes would spend, on average, almost 18 more minutes in MVPA than students in traditional instruction classes (*p* = 0.008).

#### 3.2.2. Lesson Context

Adjusting for class size, length of class, and whether a class took place indoors or outdoors, regression models predicted that FLA classes would spend significantly less class time building knowledge than traditional instruction classes: estimates for the time FLA classes would spend building knowledge were 35% lower than traditional instruction classes (*p* = 0.03). After adjusting for covariates, no significant differences emerged between FLA and traditional instruction for percent of class time spent learning general content, practicing skills, or engaging in free play.

#### 3.2.3. Teacher Involvement

The percentage of class time the PE teacher spent involved in different activities varied as a function of instructional approach. In his FLA classes and when accounting for covariates, regression models predicted that the teacher would spend 31% less time in whole group instruction (*p* < 0.01) and 58% more time observing students (*p* = 0.04) than in traditional instruction classes. No significant differences emerged in the percent of class time the teacher was involved in small group instruction, managing students, or performing other tasks.

## 4. Discussion

The purpose of this exploratory study was to compare the impact of the FLA and traditional instruction on student MVPA, lesson context, and teacher involvement using direct observation within a high-poverty, majority non-White middle school PE course. To date, limited studies have examined the use of the FLA in similar environments and lack data gathered through direct observation. Therefore, this study represents an important initial step toward understanding if and how the FLA can enhance PE to support positive, equitable MVPA opportunities and outcomes in high-poverty PE contexts. Results suggest that the FLA may be a meaningful way to increase MVPA during PE, although further research will be necessary to support or resist this claim.

The main findings of this study demonstrate the potential for the FLA to enhance MVPA opportunities during PE and diminish disparities in MVPA opportunities in PE for students in a low SES middle school [12]. Students in the FLA class engaged in significantly higher levels of MVPA over the course of a 5-lesson invasion games unit than their traditional instruction counterparts. This occurred despite the direct instruction lessons achieving above the 50% MVPA recommendations for PE lessons [43], which constituted a higher quality PE environment compared to standard PE classes globally [32,40]. Given that student MVPA levels in middle school PE are generally lower than those found in this study, especially in low SES schools, the FLA may present a significant enhancement effect across middle school contexts [12,44]. The FLA seems to have potential as an enhancement strategy with capacity to produce MVPA outcomes comparable to previous enhancement interventions, as well as more complex, multi-component interventions [31,45,46]. The results of this study are encouraging given the practicality of the intervention and point to the FLA as a feasible strategy for improving MVPA opportunities during PE. The practical nature of the FLA as a free, teacher-driven intervention sets the FLA apart from more expensive or complex research that requires extensive professional development or complicated policy change.

In addition to increasing students’ MVPA, the FLA impacted aspects of lesson context and teacher involvement. Significantly lower proportions of lesson time were spent in knowledge context and whole group instruction during class for students in the FLA class compared with the traditional instruction class, which may be how the FLA facilitated added MVPA opportunities. In other words, spending less time in knowledge context receiving whole group instruction may have opened lesson space for students to engage in more MVPA during this study. Limiting the amount of time in knowledge context during in-person classes represents a common concern related to the use of asynchronous modalities like the FLA (i.e., if FLA students do not engage with the digital content appropriately before class, there is a chance for lost knowledge, since in-person class time should be spent in applied learning rather than knowledge acquisition). There are distinct challenges with accountability when employing asynchronous digital instruction due to students’ autonomous interaction with content and the potential for diminished learning engagement [23,47]. However, in this study, students reviewed digital content during homeroom, on school-issued laptops, under the supervision of their homeroom teacher. These learning supports were possible due to school policies and PE teacher initiative to coordinate with the homeroom teacher colleague. They also helped ensure students appropriately engaged with the content prior to class and were ready for in-person applied learning. The approach outlined in this study, and similar policies, represent practical considerations for teachers and schools concerned about student accountability within FLA. Accessibility to technology and equitability of learning opportunities are also essential issues to teachers seeking to apply online (flipped) learning in PE [48,49]. The context of this study demonstrated the value of supportive digital learning environments. Resources were allotted to ensure all students who attended the school were supplied with digital learning devices (access) and corresponding policies were in place to allow time to engage with digital learning assignments (equitably opportunities). The availability of such resources and policies do not exist in many schools however, particularly those serving low-income and marginalized communities, which may represent a key barrier to FLA implementation in these contexts [50].

Less time spent in management during the FLA lessons (although results were not statistically significant) may be indicative of the prior priming students received from the digital instruction and their familiarity with lesson content and applied learning formations. While this study did not specifically examine student engagement with the digital content or analyze their learning acquisition, policies that support students’ technology-based learning are essential, particularly in subjects like PE where learning through technology may not be automatically associated with the subject [51,52].

Though differences were not statistically significant, students in the FLA classes generally participated in more skill practice opportunities than students in traditional instruction. One reason for the lack of significant findings may be the high quality of the traditional instruction, where students were already spending a higher proportion of lesson time engaged in skill practice than PE classes generally [53]. This may have limited the impact the FLA had on the amount of time students spent in skill practice but could point to the value of the FLA to improve lower quality PE. Given the emphasis on fundamental motor skill development within PE across grade-levels, further investigating the capacity of the FLA to support skill development will be an important area for future exploration.

## 5. Limitations

Despite positive indications regarding the use of FLA in PE, findings from this study should be considered within the context of its limitations as an exploratory study. First, the lesson sample size was small and limited to the content of the observed unit of instruction. Next steps for further research should test FLA with larger sample sizes and alternative contents. Ethical obligations also prevented the collection of student FLA digital content engagement data or related learning assessments, so it is unclear whether and to what extent the FLA may have impacted these outcomes. Second, the teacher in this study independently implemented the FLA with no external support other than existent, peripheral school technology policies. The positive outcomes observed during this study occurred without the teachers’ participation in professional development specific to implementing the FLA. This teacher was, however, recognized as an exemplary educator and shown to be highly effective as evidenced by the high MVPA during direct instruction lessons documented through this study. Given how teacher support has been identified as a key component to technology implementation in PE [54], as well as the successful implementation of the FLA [55], it is likely that to replicate the significant outcomes and positive indications in this study, professional development support will be necessary given the current relative novelty of the FLA in PE. Finally, this study was conducted within all boys’ PE classes and further research should extend into girls’ and mixed-gender PE contexts.

## 6. Conclusions

This exploratory study employed systematic observation to initially explore the impact of the FLA in PE. In contrast to other interventions that rely on robust professional development and/or more complex strategies, this study showed that the FLA may be a practical strategy to enhance MVPA outcomes in PE within a high-poverty, low-income school and could address a lack of equity in MVPA opportunities in middle school PE. It showed that opportunities for digital learning engagement and greater in-class participation in MVPA during PE lessons are possible within the FLA versus traditional instruction class. This study also gives reason to support a broader conceptualization of *enhancement* within the TEO [31] to include the use of the FLA to improve existing PE opportunities. Given the preliminary nature of this research, it will be important to broaden the scope of study in this area to gain more substantial insights into the utility of the FLA and its ability to promote positive outcomes within PE, across a variety of learning environments. Nevertheless, results indicate the FLA warrants further study given its potential as an enhancement strategy to increase equitable MVPA and learning opportunities in middle school PE.

## Figures and Tables

**Table 1:** Descriptive Statistics of SOFIT Variables of Interest in Physical Education Classes Using Flipped Learning and Direct Instruction Approaches.

		Flipped			Direct	
	N	M (SD)	Range	N	M (SD)	Range
Class Size (# of students)	5	28.60 (1.14)	27.00–30.00	5	24.80 (1.64)	22.00–26.00
Length of Class (min)	5	32.00 (2.12)	29.00–35.00	5	32.40 (1.95)	30.00–35.00
Indoor Classes	2			2		
Outdoor Classes	3			3		
**PA Engagement**						
MVPA	5	74.84 (14.85)	52.30–88.30	5	53.12 (15.23)	29.70–70.30
MVPA (min)	5	24.16 (6.01)	15.10–30.50	5	17.20 (5.07)	9.00–21.70
**Lesson Context**						
General Content	5	24.94 (11.07)	8.00–36.80	5	28.84 (6.14)	21.00–35.80
Knowledge	5	2.26 (3.55)	0–8.10	5	23.58 (16.93)	9.90–48.40
Skill Practice	5	55.02 (30.94)	0–72.00	5	38.6 (25.07)	0–58.00
Free Play	5	17.60 (39.35)	0–88.00	5	9.00 (20.12)	0–45.00
**Teacher Involvement**						
Instructs Small Group	5	30.28 (25.22)	0–58.10	5	19.04 (10.71)	8.80–36.80
Instructs Whole Group	5	3.12 (3.38)	0–8.10	5	15.28 (10.72)	8.40–34.10
Manages	5	30.72 (12.11)	17.40–48.00	5	43.08 (3.77)	37.00–47.30
Observes	5	31.54 (21.47)	10.50–63.80	5	20.30 (14.59)	2.20–35.00
Other Tasks	5	4.34 (3.54)	0–9.50	5	2.06 (2.15)	0–5.00

Note. PA = physical activity; MVPA = moderate-to-vigorous physical activity. All SOFIT variables presented as % of class time unless otherwise noted. Three SOFIT variables (fitness activity, teacher promotes fitness, teacher demonstrates fitness) did not occur in observed sessions.

**Table 2:** Results of Individual Linear Regression Models Predicting Each SOFIT Variable by Instructional Style (Flipped Learning vs. Direct Instruction Approach).

SOFIT Dependent Variables	β (95% Confidence Interval)	SE	*p*-Value
**PA Engagement**			
MVPA	55.03 (21.30, 88.75)	13.12	<0.01
MVPA (min)	17.70 (6.90, 28.49)	4.20	<0.01
**Lesson Context**			
General Content	−7.80 (−27.78, 12.18)	7.74	0.36
Knowledge	−34.89 (−63.34, −6.43)	11.07	0.03
Skill Practice	−15.97 (−80.20, 48.25)	24.98	0.55
Free Play	58.22 (−24.93, 141.37)	32.35	0.13
**Teacher Involvement**			
Instructs Small Group	−22.72 (−94.12, 48.69)	27.78	0.45
Instructs Whole Group	−31.30 (−47.66, −14.95)	6.36	<0.01
Manages	−6.91 (−37.57, 23.75)	11.93	0.59
Observes	58.30 (5.64, 110.96)	20.48	0.04
Other Tasks	2.51 (−9.32, 14.35)	4.60	0.61

Note. PA = physical activity; MVPA = moderate-to-vigorous physical activity. In each model, direct instruction classes served as the reference group (coded as 0); flipped instruction classes were coded as 1. Each model adjusts for class size, length of class, and whether class took place indoors or outdoors. All SOFIT variables assessed as % of class time unless otherwise noted.

## Data Availability

Not applicable.
